# The Physics of DNA Folding: Polymer Models and Phase-Separation

**DOI:** 10.3390/polym14091918

**Published:** 2022-05-09

**Authors:** Andrea Esposito, Alex Abraham, Mattia Conte, Francesca Vercellone, Antonella Prisco, Simona Bianco, Andrea M. Chiariello

**Affiliations:** 1 Dipartimento di Fisica, Università di Napoli Federico II, INFN Napoli, Complesso Universitario di Monte Sant’Angelo, 80126 Naples, Italy; andresposito@na.infn.it (A.E.); abraham@na.infn.it (A.A.); matconte@na.infn.it (M.C.); francesca.vercellone@na.infn.it (F.V.); 2 CNR-IGB, Via Pietro Castellino 111, 80131 Naples, Italy; antonella.prisco@igb.cnr.it; 3 Berlin Institute for Medical Systems Biology, Max-Delbrück Centre (MDC) for Molecular Medicine, 10115 Berlin, Germany

**Keywords:** phase-separation, chromatin organization, polymer physics, gene regulation, molecular dynamics, phase transitions

## Abstract

Within cell nuclei, several biophysical processes occur in order to allow the correct activities of the genome such as transcription and gene regulation. To quantitatively investigate such processes, polymer physics models have been developed to unveil the molecular mechanisms underlying genome functions. Among these, phase-separation plays a key role since it controls gene activity and shapes chromatin spatial structure. In this paper, we review some recent experimental and theoretical progress in the field and show that polymer physics in synergy with numerical simulations can be helpful for several purposes, including the study of molecular condensates, gene-enhancer dynamics, and the three-dimensional reconstruction of real genomic regions.

## 1. Introduction

In the past two decades, the development of advanced experimental techniques, such as Hi-C and super-resolution microscopy, that map the spatial genomic architecture [1,2] highlighted the deep link between genome structure and vital cellular functions such as transcription, replication, and DNA repair [3]. Indeed, for example, gene expression can be controlled by physical contacts occurring between the gene promoter and specific, distal DNA regulatory regions such as enhancers [4]. Notably, genomic structural variants, e.g., deletions or duplications, have been found to disrupt regulatory contacts between genes and enhancers, thus resulting in severe human diseases [5]. Furthermore, these novel experimental tools revealed the presence of complex, non-random features of chromatin three-dimensional (3D) conformation [6] across length scales. At the megabase scale, chromosomes are organized in a sequence of topologically associated domains (TADs), characterized by strong internal interactions [7,8]. At the same scales, strong loops are also observed in the contact maps between distal DNA sites, such as pairs of convergent CTCF binding sites or gene-enhancer pairs [9]. Non-trivial contact patterns have been also observed at the sub-TAD level [10] and at larger scales where TADs can interact with each other in higher-order structures, named meta-TADs [11], thus forming, together with TADs and sub-TADs, a hierarchy of domains within domains. At larger scales, 10 Mb-sized A/B compartments of active/repressive chromatin have been discovered [12]. Other important features of the genome organization within the cell nucleus include the formation of multiple contacts, e.g., triplets, between distal DNA sites such as super-enhancers [13], non-trivial inter-chromosomal contacts around the nucleolus and nuclear speckles [14] and so-called lamina-associated domains (LADs), repressed domains interacting with the nuclear lamina [15]. Moreover, DNA folding is found to be highly dynamic, variable across single cells in a population [16] as well as through the cell cycle [17,18] and cell differentiation [11,19].

In parallel, intense research has been conducted to understand the biophysical mechanisms that underlie this organization and allow the correct activity of the cell. Phase-separation, i.e., the formation of membraneless aggregates through interaction between molecules [20,21], turned out to be a fundamental mechanism associated with gene regulation and chromatin structure. Phase-separated condensates can form both in vitro and in vivo [20] and within cells, they can be observed at different scales. Important examples are nucleoli and the Cajal body [20,21] or droplets of coactivators such as BRD4 and MED1 [22] and of transcription factors [23]. Recently, it has been shown that protein chimeras associated with leukemia form clusters through phase-separation [24]. Furthermore, many more studies highlight different features of phase-separated aggregates [25,26,27,28,29,30]. Along with experimental research, polymer models were also developed to quantitatively understand the principles shaping chromatin structure. Interestingly, several approaches naturally envisage the need for phase-separation mechanisms [31,32,33,34,35,36,37] to exhaustively explain multiple experimental observations. Here, we first give a brief review of some recent theoretical and computational approaches showing the versatility of polymer models to tackle the problem of chromatin folding in general and to study the underlying molecular processes. We then discuss how basic models [35] systematically investigate the phase-separation of protein aggregates and their relationship with gene regulation in a simplified, yet highly controlled framework. Then, using as case studies the murine α-globin and HoxD genomic regions [38,39], we show that the models informed with experimental data are able to accurately describe the 3D organization of real genomic loci and to study complex architectural rearrangements occurring during cell differentiation. Importantly, in these examples, the physical mechanism that confers the structural features to these regions is a thermodynamically driven micro-phase separation condensation of molecules, triggered by their interaction with the polymer. Finally, we briefly discussed the role of additional mechanisms alongside phase separation, such as the loop-extrusion, in order to comprehend the physics of chromatin organization.

## 2. Polymer Models of Chromatin Organization

To investigate chromatin folding, two main classes of models exist: top–down (also known as data-driven) and bottom–up models. Within the former, numerical and computational strategies are employed to infer the 3D structure of a specific genomic region directly from the data, typically obtained from 3C-based methods [40,41,42,43,44,45], without taking into account specific physical processes. Here, chromatin is modeled as a polymeric chain of beads, and a cost function linking the data in the input to the positions of the beads in 3D space, which is used to optimize spatial constraints such as excluded volume effect and polymer stiffness. Such a strategy has been successfully applied to a variety of problems including single-cell data [46], structural rearrangement during time [47], modeling RNA–DNA interactions [48], structural models of diploid genomes [49,50]. Conversely, in the bottom–up (known also as first-principled) scenario, polymer-physics-based models are used. Here, the molecular mechanisms shaping the DNA 3D structure are imposed a priori and model predictions are compared with experiments. Importantly, these kinds of models are clarifying many architectural features emerging from experimental data (such as HiC [12], GAM [13], Sprite [14]), as well as the specific molecular mechanisms that spatially organize different loci. In some of these models, contacts between distal DNA sites are induced by diffusing molecules, e.g., transcription factors (TFs) [31,32,33,51,52], which are known to bind DNA [53,54,55], or by direct interaction between epigenetically similar genomic regions [56]. Here, phase-separation is the fundamental driving mechanism that shapes the chromatin structure and establishes contacts between regulatory elements. Other models consider the extrusion of chromatin loops [57,58,59] by active molecular motors such as cohesin, which has been experimentally observed [60,61]. More recently, models combining both phase-separation and loop-extrusion have been developed [62,63]. A more complete description of the main polymer physics models for chromatin can be found in [64,65,66]. In the following sections, we focused on the Strings&Binders Switch (SBS) model, recently used to study several genomic regions in wild-type genomes, to explore chromatin’s structural variability at the single-cell level and to predict the impact of disease-linked mutations [38,39,52,67,68,69].

## 3. Phase-Separation and Gene-Enhancer Contact Dynamics

**Figure 1:**
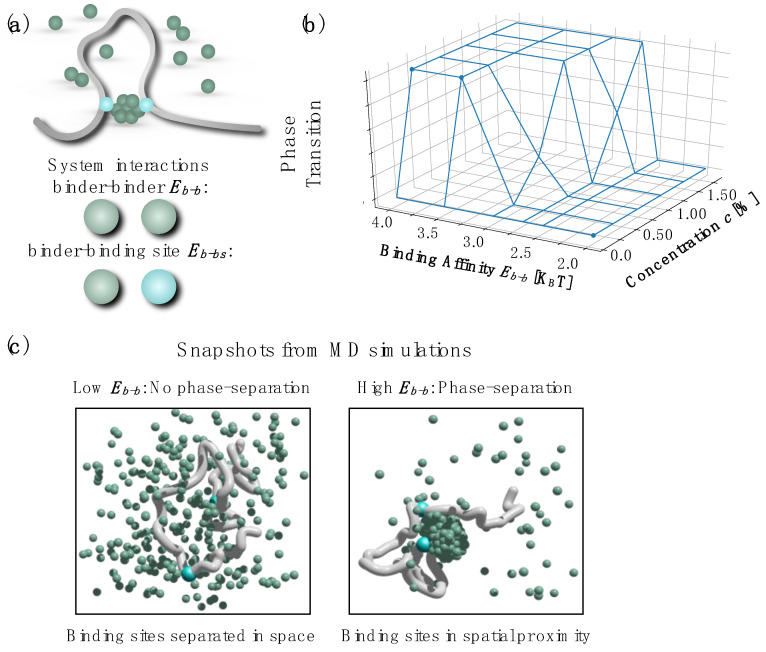
Polymer models and phase-separation in cell nucleus. (**a**) In the SBS model, the polymer chain has binding sites that can interact with diffusing molecules (binders) with affinity E_b-bs_. Binders can also self-interact, with affinity E_b-b_, so to drive the formation of phase-separated clusters. (**b**) Phase-diagram of the system as a function of binder concentration and binder–binder interaction affinity E_b-b_. Red indicates the formation of the phase-separated cluster; and blue indicates that the cluster is not formed. (**c**) Three-dimensional snapshot of the system without (blue) and with (red) phase-separated cluster. If formed, the phase-separated cluster mediates the contact between binding sites. Panels adapted from [35].

In the SBS model [31,32,70], chromatin is a self-avoiding chain of beads with some binding sites that can interact with molecular factors (binders) dispersed in the surrounding environment. In general, each binding site has a specific type of interaction (or color) and can experience an attractive potential only with its cognate binders in a homotypic fashion [71] (Figure 1a). The binders stably bridge their cognate sites, driving the folding of the system through a phase-separation-based process. To also model the phase-separation of protein, typically mediated by the self-interaction among their intrinsically disordered domains (IDR) [20,21], an interaction among binders can be introduced [35] according to the schematic in Figure 1a. The controlling parameters of the system are therefore: binder concentration, binding site–binder affinity (E_b-bs_) and binder–binder affinity (E_b-b_). By use of extensive Molecular Dynamics (MD) simulations, the system can be explored under different conditions as shown in the phase-diagram of Figure 1b: a phase-separated cluster of binders can form only if the concentration and E_b-b_ are above the transition threshold (E_b-bs_ kept fixed) [35], otherwise, the cluster is not observed. Importantly, the contact between the distant binding sites on the polymer is mediated by the formation of the phase-separated cluster (Figure 1c), in analogy with recent studies linking the contact between the enhancer (or super-enhancer) and genes with the phase-separation of proteins [24,26,72].

**Figure 2:**
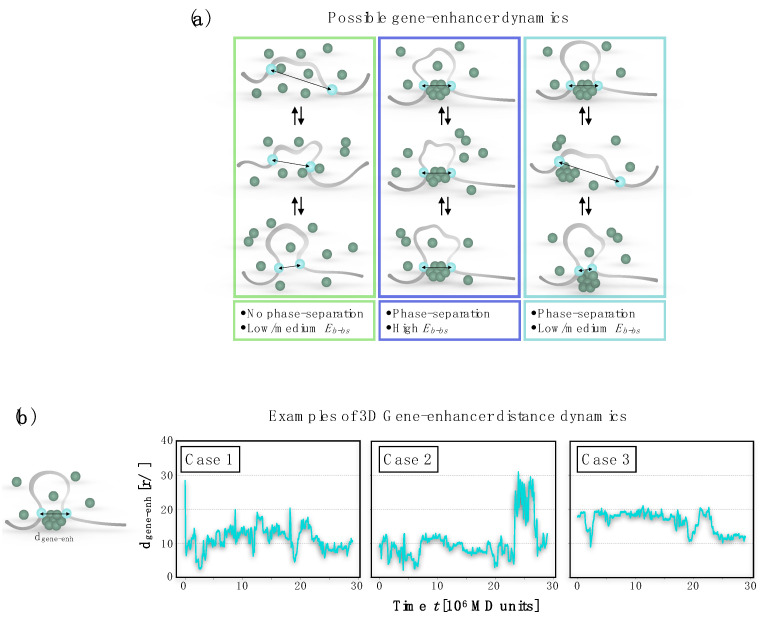
Dynamic of the contact between regulatory elements. (**a**) The affinity between binding sites and binders influences the contact during time and different dynamics emerge. (**b**) Different examples of contact dynamics obtained from Molecular Dynamics simulations, for a moderate affinity E_b-bs_ = 3.1 KBT (E_b-b_ = 3.0 KBT). The contact is stable, but the binding sites can move on the cluster and can also detach. Adapted from [35].

An interesting, general aspect of this modeling approach is the possibility of quantitatively investigating several features associated with gene regulation in a highly controlled way. An important example is the study of the dynamics between regulatory elements, which can be easily explored in this framework. Indeed, by tuning the binding site-binder affinity E_b-bs_, it is possible to regulate the dynamics regime of the contact between the binding sites according to the scheme shown in Figure 2a. Three main behaviors emerge. With low interactions affinities and low concentrations, no phase-separated cluster can form and, consequently, no stable contact is possible between the binding sites (Figure 2a, left panel). Conversely, if the phase-separated cluster of binders is formed and the interaction with the binding sites on the polymer is very strong, the resulting contact is very stable and the associated dynamics is practically flat (Figure 2a, central panel). Finally, if the cluster is formed and the affinity between binders and binding sites is moderate, a contact between the binding sites is formed but it is more sensible to the thermic fluctuations, as the binding sites can move on the cluster or can detach from that. Therefore, the resulting contact dynamics appear more variable (Figure 2a, right panel). The temporal dynamics of the contact can be easily studied by monitoring the spatial distance between the binding sites obtained from MD simulations of the model. For example, Figure 2b shows some possible distance dynamics with moderate interaction affinities between the binders and binding sites. Importantly, such simulated temporal dynamics can be compared with experiments, as recently performed with real-time imaging data [73] tracking the distance between the Sox2 gene with its super-enhancer in different cell types [35]. This opens the way, once more experimental data are available, to test the mechanisms governing the dynamics of regulatory elements in real genomic regions.

To conclude this section, we mention that other important physical features can be quantitatively investigated using this kind of models. These include the equilibrium conformations of the system, its cell-to-cell structural variability and more generally, the microscopic processes underlying the formation of the phase-separated aggregate. Those include mechanisms of the initiation of the cluster (e.g., nucleation) and the evolution of cluster structure (driven, e.g., by evaporation and coalescence) [35], which are also object of intense experimental research [20,22,24,26].

## 4. Polymer Models and Real Genome Reconstruction

**Figure 3:**
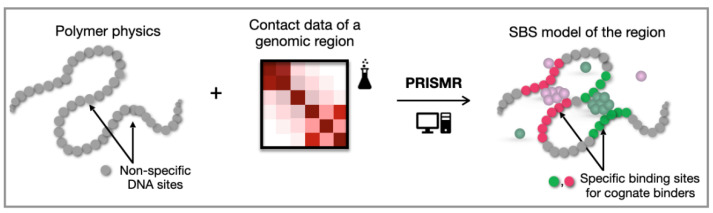
PRISMR and machine learning of 3D genome conformations. PRISMR is a machine learning-based procedure which, informed by a polymer physics model of chromatin and experimental pairwise contact data of a given genomic region of interest, infers the optimal polymer model of the region (adapted from [75]).

By considering more complex polymer models, it is possible to reproduce the folding of real genomic regions, having specific, intricate contact patterns as seen, e.g., in Hi-C or GAM experiments. To this aim, it is necessary to specialize the polymer model, i.e., the attractive interaction between the polymer and binding molecules, by introducing different types of the polymer sites, each interacting with their specific cognate binders. One way to specialize the polymer sites for a given genomic locus is the use of available chromatin marks, such TFs binding sites or histone modifications, as performed, e.g., in [55,62]. As an alternative approach, it is possible to infer the polymer binding sites based on experimental contact data. Interestingly, this second approach is not limited by use of previously known chromatin binding sites and has the potential to discover novel chromatin organizing factors. Following this approach, a machine learning-based procedure [71] was recently developed, named PRISMR (polymer-based recursive statistical inference method). PRISMR takes the experimental contact data as input, e.g., Hi-C [71] or GAM [74], of the region of interest, and returns as output the optimal polymer model, i.e., minimal number of different types of binding sites (colors) and their arrangement along the polymer, whose 3D folding at the thermodynamic equilibrium best explains the input data (Figure 3). PRISMR was successfully used to study the architecture of several chromatin regions, including EPHA4, Pitx1, Shh, and HoxD [39,67,68,69,75] and recently extended to model entire genomes [76]. In addition, the impact on the 3D architecture of large structural variants associated with human diseases was explored [71], and other features of chromatin folding, such as structural variability at the single-cell level [52], were successfully predicted with independent validation, e.g., microscopy data [16]. In the following, we illustrate example applications of such an investigation approach in two case studies, the α-globin and HoxD loci.

### 4.1. The α-Globin Locus: Structural Reorganization upon Differentiation

**Figure 4:**
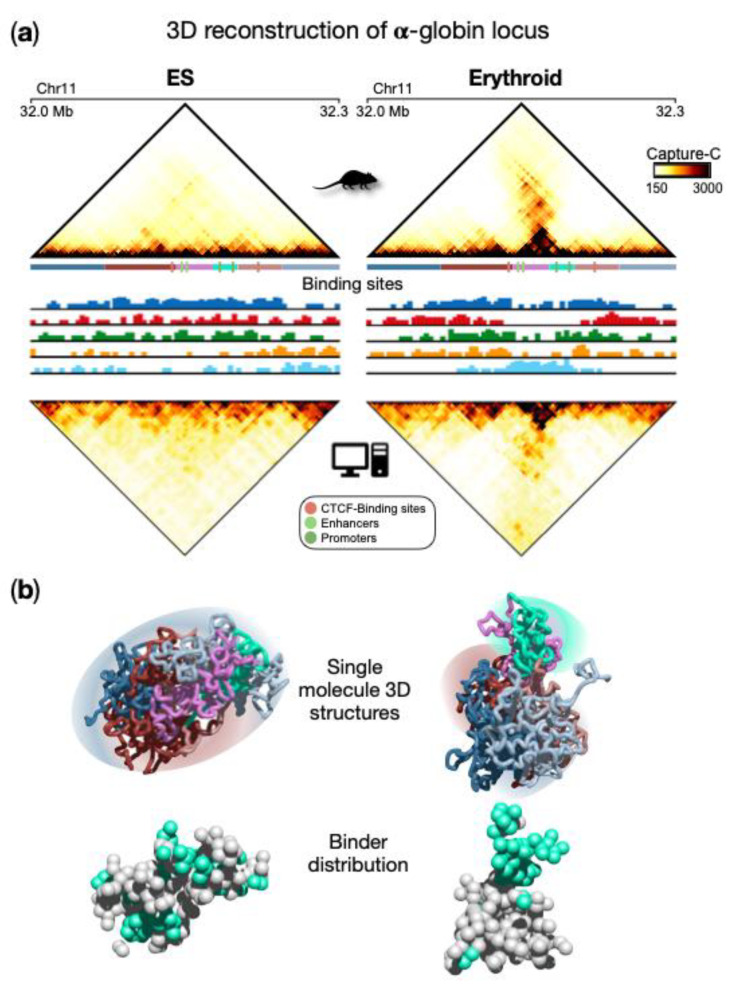
Polymer physics highlights regulatory linked conformations driven by micro-phase separation in α-globin locus. (**a**) Upper panels: Capture-C contact data of the genomic region centered on the α-globin genes show a drastic change in the 3D organization of the locus in ESCs with respect to erythroid cells, where a strong stripe of interaction emerges. Bottom panels: contact data obtained from the ensemble of 3D conformations from Molecular Dynamics simulations of the SBS model; (**b**) Examples of single-molecule 3D structures in the two cell types highlight chromatin conformational tissue-specificity: in ESCs, a large uniform domain with no preferential contact is observed, while in erythroid, evident domains form, one containing the globin genes and enhancers. Binder distributions are shown next to the polymer structure, binders associated with genes and enhancer are colored in cyan. Image adapted from [38].

Here, we briefly show a recent application of the SBS model to reconstruct the three-dimensional structure of real genomic loci. As a case of study, we focus on a region centered around the α-globin genes in mouse ESCs and primary erythroid cells [38], where high-resolution Capture-C data have been employed [67] to study its 3D organization. These data, which refer to a 300 kb-sized genomic region, highlight drastic architectural rearrangements in the transition from ESCs to erythroid (Figure 4a, upper panels). Specifically, in ESCs, contacts are rather homogeneously distributed within the locus in a uniform pattern, and the globin genes are silent. In erythroid cells, a prominent self-interacting domain, containing genes and enhancers, is visible and extends in the matrix along the opposite diagonal. This structure is functional to the activation of the globin genes in erythroid cells [38,67]. By applying the PRISMR procedure to the Capture-C data, SBS polymer models were inferred for both ESCs and erythroid cells (Figure 4a). Interestingly, the inferred different types of binding sites were found to correlate with specific combinations rather than single chromatin marks [38]. By running MD simulations of the obtained polymer models (see [38] for technical details), a set of 3D single-molecule conformations were produced and accurately recapitulate the contact data of this locus in both cell types (Figure 4a lower panels). The comparison between Capture-C and simulated contact maps (Figure 4a) shows that most of the experimental patterns were well reproduced by the SBS model, as also highlighted by high Pearson correlation coefficients (Pearson r = 0.96 in both ESCs and erythroid). This is an indication that the model 3D structures could represent the real architecture of the region to a good extent and support a principled interpretation of the observed contact patterns based on polymer physics. For example, TADs can be interpreted as regions enriched for contacts between specific types of binding sites, which biologically correspond to specific combinations of chromatin marks. More specifically, from a quantitative analysis of the obtained conformations [38], two different types of topologies emerge for ESC and erythroid cells, as also highlighted by single molecule snapshots (Figure 4b). In ESCs, a rather uniform structure is visible with high intermingling among different parts of the locus, while in erythroid structures, more discrete interaction domains appear and are poorly intermingled, consistent with Capture-C data. It is important to note that such structural properties predicted by the model emerge from the interactions between the binding sites of the polymer and the binders, whose distribution is shown in Figure 4b. This eventually leads to the micro-phase separated clusters of binders associated with the interaction domain observed in erythroid cells, but not in ESCs (Figure 4b). Beyond the interactions producing the pairwise contacts observed in this locus, such a mechanism allows the formation of high-multiplicity regulatory contacts between genes and enhancers, as discussed in the next section.

### 4.2. Micro Phase-Separation Leads to High-Multiplicity Regulatory Contacts at the α-Globin Locus

**Figure 5:**
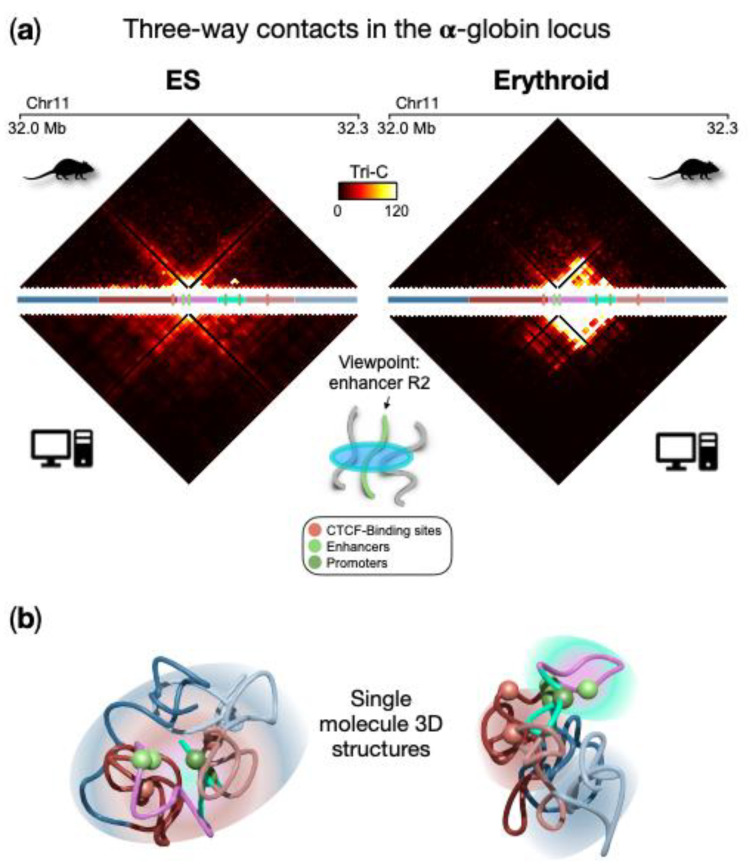
High-multiplicity regulatory contacts at the α-globin locus. (**a**) Three-way contact probability from the ensemble of polymer conformations in ESCs and erythroid (upper panel) and from the TriC [67] experimental method (bottom panels), with the enhancer R2 as a point of view; (**b**) Example of a single molecule 3D structure obtained from Molecular Dynamics simulations highlights the structural features in ESCs and erythroid cells. Spheres indicate the positions of relevant regulatory elements. Image adapted from [38].

An important aspect of the discussed approach is that we can extract more general, quantitative information about the specific architecture from the ensemble of 3D structures, beyond the simple pairwise contact probability. A natural extension is represented by multi-way contacts, i.e., contacts among more than two loci (e.g., triplet if three loci, quartets if four loci, etc.). Here, we focus on the three-way contacts (triplets) of regulatory sites (genes and enhancers) that have been predicted from the polymer model and compared with experimental data obtained with the recently developed Tri-C technique [67]. Without loss of generality, we consider as a point of view the strongest enhancer (named R2) for the α-globin genes and calculate the probability of a triplet with any other pair of loci within the locus, in ESCs and erythroid cells. As shown in Figure 5a (upper panels), the two cell lines exhibit different features. Indeed, in ESCs there is no specific three-way interaction pattern, in agreement with its 3D architecture where the polymer is highly intermingled and only proximity effects dominate. Conversely, in erythroid, a non-random pattern of triplet contacts emerges, with a high probability of multi-way contacts localized in the region spanning the enhancers and the globin genes. Interestingly, this is compatible with the hairpin structure proposed as a description for this locus in erythroid cells [38] and driven by the polymer-binders phase-separation mechanism we previously discussed. In this structure, the region containing genes and enhancers spatially segregates and weakly intermingles with its flanking regions, which in turn tend to be more intermingled and in close spatial proximity. As the validation of these results, the prediction of three-way contact probability was compared with independent experimental data generated by the Tri-C method (Figure 5 bottom panels), which measures the probability of a triplet for a fixed point of view. Good agreement between the model and experimental data confirms the validity of the prediction for enhancer R2, also highlighted by the high correlation coefficient (Pearson r = 0.8 in both ESCs and erythroid cells [38]). An analogous agreement is obtained by considering as a point of view a CTCF site located upstream the gene region (average correlation coefficient r = 0.8). Single snapshot 3D conformations obtained from real Molecular Dynamics simulations highlight such higher-order contacts and are helpful to visually capture the above-described structural features in ESCs and erythroid (Figure 5b).

It is important to note that the ensemble of 3D conformations is only derived from pairwise experimental matrices and the probability of such triplets is computed purely from the geometric coordinates in the simulated 3D structures. Therefore, this kind of analysis is general and can be applied to other loci for which a population of three-dimensional structures has been inferred, as shown in the following section. Furthermore, the method can be used to calculate higher-order contacts involving more than three objects [34].

### 4.3. High-Multiplicity Regulatory Contacts at the HoxD Locus

**Figure 6:**
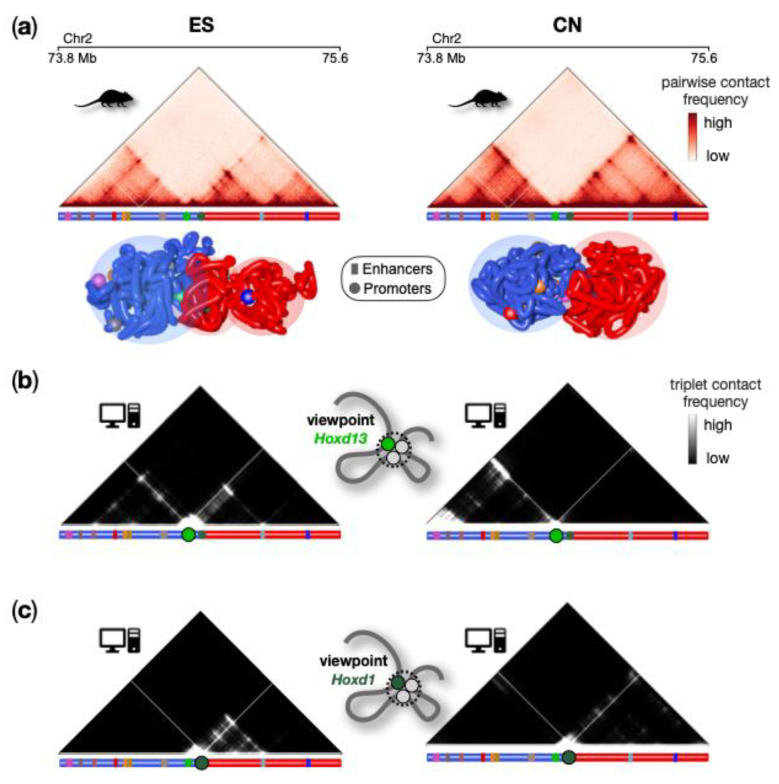
Polymer physics highlights high-multiplicity regulatory contacts at the HoxD locus. (**a**) High-resolution Hi-C data [19] of the HoxD genomic region and model derived single-molecule 3D conformations in ES (**left**) and CN (**right**) cells help visualize the locus structure with its two flanking TADs in the two cell types. (**b**,**c**) Model predicted triple contact frequency form the viewpoint of HoxD13 (**b**) and HoxD1 (**c**) have a gene and cell-type-specific organization. Image adapted from [39].

Here, we illustrate the modeling of a region around the murine HoxD genes clusters, which is involved in the formation of animal body structures during embryo development [39,77,78]. Two cell types, ESCs and cortical neurons (CNs), were considered in order to explore the architectural changes occurring when the HoxD genes change from a poised to a silent transcription state [39]. To infer polymer models for this region, PRISMR was applied to high-resolution (5 kb) Hi-C data [19] (Figure 6a) and ensembles of 3D conformations were obtained by MD simulations. In Figure 6a, example snapshots from the model 3D structures are shown in ESCs and CNs cells, with the positions of key genes and regulatory regions highlighted. Interestingly, however, the 3D models show a strong structural variability in both ESCs and CNs. This is highlighted, e.g., by the standard deviation to the average ratio of the distance between HoxD1 and HoxD13 (the rightmost and leftmost gene in the cluster, respectively), which is approximately 30%. At the same time, there is high cell-type specificity in the 3D structure, as, e.g., the HoxD13 and HoxD1 genes are found approximately 50% further in CNs, showing that the poised HoxD cluster in ESCs is more compact than in CNs. As before, the corresponding average pairwise contact matrices from the 3D models well reproduce the Hi-C contact pattern correlations (see [39] for details). Beyond pairwise contacts, the model has the ability to predict further structural features such as the formation of high-multiplicity, many-body contacts, information that can be easily derived from reconstructed 3D ensembles. In this way, abundant triple contacts were discovered, involving promoters and regulatory regions that are highly gene and cell-type specific (Figure 6b,c). As an example, in Figure 6b, the triplets formed by Hoxd13 gene with the rest of the locus are shown, while in Figure 6c, the triplets from the Hoxd1 viewpoint are shown. Importantly, in ESCs, independent 3-way 4C data are available [79] that have been used to validate the model predictions of triplet contacts from several viewpoints [39]. Interestingly, the model-derived triple contact probability from the different viewpoints has a gene- and cell-type-specific compartmentalized structure. The triplets formed by HoxD13, for example, are mostly restricted within the TAD downstream the HoxD cluster, in both ESCs and CNs. On the contrary, the triplets formed by HoxD1 are mainly within the downstream TAD in both cell types. Additionally, gene-specific subsets of triplets are formed by genes and their regulatory regions [39]. This indicates that such combinations of higher-multiplicity interactions are highly selective and suggest that they can be the mode of action whereby the 3D conformation differentially regulates the genes. Taken together, the polymer modeling results helped to shed light on the organization of the murine HoxD region, which is characterized by a network of cell-type and gene-specific many-body contacts with regulatory elements and undergoes deep reorganization upon differentiation from ESCs to CN cells in parallel with the changes in the activity of the genes.

In the last sections, we review specific applications of polymer physics models to describe different features of the three-dimensional architecture for some real genomic loci. In those examples, the computational procedure was applied on experimental data of very short genomic regions. However, it is worth mentioning that the same approach was recently extended genome-wide in order to identify the location of the putative binding sites underlying DNA–DNA interactions and to derive a first characterization of their molecular features [76]. In particular, the combination of the architectural information (3D contacts) with the genomic position of the features determining the state of the chromatin (1D epigenetics), provides a code to successfully predict de novo, from only epigenetics, the 3D structure of independent chromosomes [76].

## 5. Further Mechanisms of DNA Folding: The Loop-Extrusion Model

As illustrated above, polymer models based on phase-separation processes such as the SBS can explain many key features of DNA folding, from TADs to complex higher-order interactions. However, they fail to explain other experimental observations, such as the so-called CTCF convergence bias, i.e., the property that loops between CTCF sites are predominantly formed by convergent CTCF motifs. A different class of models based on a loop-extrusion (LE) mechanism can, however, easily explain these observations. In LE models [57,59], DNA folding is driven by a DNA tracking mechanism: a loop extruding factor (LEF) anchors at a chromatin site and then actively extrudes a loop until stopped by some barriers present along the chromatin. In particular, the cohesin complex has been proposed as LEF and DNA sites bound by CTCF proteins as the halting barriers. Notably, the CTCF convergence bias is easily reproduced by requiring that only convergent CTCF sites can halt cohesin. In the LE, the extrusion is assumed to be an ATP-driven process. A variant of this model was also developed, the slip-link (SL), that consider cohesin sliding over the DNA by thermal diffusion, without an active, energy burning motor [58,80]. LE based models can explain a number of architectural features such as loops and stripes detected in experimental contact maps. However, they also have a number of limitations. Indeed, recent microscopy experiments showed that while cohesin depletion abolishes TADs at the population average level, TADs are preserved in single cells upon cohesin depletion, suggesting that LE may drive preferential TADs boundaries’ positions rather than being necessary for their formation. Furthermore, LE-based models cannot explain A/B compartmentalization, as well as the formation of recently observed regulatory hubs involving high multiplicity contacts between genes and regulators, all features that are easily reproduced by the phase-separation models discussed above. Interestingly, these observations led to the recent proposal of hybrid models combining both mechanisms, i.e., phase-separation and loop-extrusion. However, understanding their range of action and their precise interplay, for example, if they are competitive or cooperative molecular processes, remain open questions.

## 6. Discussion

The improvement of experimental technologies [12,13,14,16,81] along with the development of polymer models are unveiling the complexity of chromatin structure in the cell nucleus and the underlying physical mechanisms acting at the molecular level. Among those, phase-separation, a well-known physical process by which protein condensates spontaneously form [20,21], plays a crucial role. Here, by using simple polymer physics models [31,32,35] and intensive Molecular Dynamics simulations, we showed a quantitative description of the phase-separation process leading to the formation of molecular aggregates. Analogously, the link of such a process with the formation of stable contacts between regulatory elements, confirmed by recent experimental evidence [22,24,26], can be effectively studied [35]. In addition, we showed that such models enable one to accurately describe the spatial architecture of real genomic regions, using the α-globin and the HoxD loci as examples. In this scenario, the peculiar structure exhibited by the α-globin locus in erythroid cells originated from a mechanism of micro phase-separation allowing the formation of higher-order contacts that facilitate the spatial proximity of genes and enhancers.

Beyond phase-separation, other biophysical mechanisms were also found to play an important role in shaping chromatin architecture, such as loop-extrusion. LE can in particular easily explain the formation of loops and stripes. However, the precise interplay between the two different mechanisms remains to be investigated. It is also possible that additional mechanisms have to be considered to explain finer aspects of chromatin folding at all length scales, as they are emerging from rapidly evolving experimental techniques returning chromatin contacts at increasingly higher resolutions across genomic regions, cell types and organisms. Finally, more realistic models are also expected to take into account finer complications such as crowding effects and volume confinement due to the nuclear envelope and nuclear bodies.

Taken together, the discussed results highlight how theoretical approaches such as models from polymer physics and numerical simulations, are valuable tools to quantitatively investigate the molecular mechanisms underlying chromatin folding and, more generally, the link between genome organization and the processes occurring in the cell nuclei. Improvements of those models will include finer aspects of chromatin folding, e.g., the combination of multiple mechanisms as phase-separation and loop-extrusion and the effect of spatial confinement and molecular crowding.

## Data Availability

Not applicable.
